# Performance of Biodegradable Biochar-Added and Bio-Based Plastic Clips for Growing Tomatoes

**DOI:** 10.3390/ma15207205

**Published:** 2022-10-16

**Authors:** Krystyna Malińska, Agnieszka Pudełko, Przemysław Postawa, Tomasz Stachowiak, Danuta Dróżdż

**Affiliations:** 1 Faculty of Environment and Infrastructure, Częstochowa University of Technology, Częstochowa, Brzeźnicka 60A, 42-200 Częstochowa, Poland; 2 Faculty of Mechanical Engineering and Computer Science, Czestochowa University of Technology, Armii Krajowej 19c, 42-200 Częstochowa, Poland

**Keywords:** biodegradable plastics, biochar, biodegradation, composting, horticulture, stem support clips, arch support clips

## Abstract

Increasing quantities of waste from using conventional plastic in agriculture and horticulture is one of the most pressing issues nowadays. Conventional plastic accessories (e.g., mulching films, clips, pots, strings, etc.) are typically fossil-derived, non-biodegradable and difficult to recycle after their use. Therefore, there is a need for biodegradable and bio-based alternatives with similar properties to conventional plastics, which can be disposed of through degradation in water, soil or compost under specific conditions. This work investigated the properties and the performance of biodegradable biochar-added and bio-based stem and arch support clips. In addition, the investigated clips were composted with tomato residues during 16 week laboratory composting. The scope of this work included: (1) the production of stem and arch support clips in a pilot installation using injection molding technology, (2) an analysis of their chemical composition, biodegradability, disintegration and phytotoxicity, (3) an evaluation of their performance in the greenhouse cultivation of tomatoes and (4) an evaluation of the composting of the clips with on-farm organic waste as an end-of-waste management method. The stem support clips during industrial composting (58 °C) degraded at 100% after 20 weeks, whereas during home composting (30 °C) the degradation was slow, and after 48 weeks the maximum weight loss was 5.43%. Disintegration during industrial composting resulted in 100% fragmentation into particles with sizes less than 2 mm. Phytotoxicity tests demonstrated that the substrates after industrial and home composting did not have a negative effect on the growth of the test plants (i.e., mustard, wheat, cuckooflower). The biochar-added stem support clips proved to be satisfactory alternatives to conventional non-biodegradable, fossil-derived clips and can be disposed of through composting. However, more work is needed to determine the optimal conditions for composting to ensure rapid degradation of the clips in relevant environments.

## 1. Introduction

Nowadays, non-biodegradable, fossil-derived plastics (often referred to as conventional) are considered a major environmental challenge. This is primarily due to the depletion of fossil fuels to produce polymers, the increased generation of plastic waste, and, as a consequence, the contamination of the natural environment with microplastics. In particular, the contamination of soil with microplastics is considered a serious threat and has been extensively reported in the literature [1,2]. The most common microplastic contaminants detected in agricultural soil include non-biodegradable, fossil-derived polyethylene (PE), polypropylene (PP) and polyvinyl chloride (PVC). These conventional plastics can persist in the environment for a very long time. Degradation times depend on a product’s characteristics (e.g., clips, pots, covering and mulching films) and the type and conditions of the environment (e.g., soil, marine, compost, landfill) [3]. Microplastics can cause reduction in soil aggregation and soil bulk density, and increase the evaporation rate of soil water. Depending on the type, dose, shape and particle size, these microplastics can have an effect on soil properties, and microbial and plant systems [4]. 

The highest consumption of plastics is reported to be for packaging (40.5%) and building (20.4%). In agricultural applications the use of plastics is about 3.2% (44% in plant production and 56% in animal production) [5]. However, the use of plastic materials such as plastic mulching, cover and silage films, clips, nursery pots, strings and ropes, etc., is gradually increasing. For example, the use of plastic mulching films is expected to increase by 59% to 2026 [6,7]. The average annual consumption of plastics in agricultural applications is estimated at about 4.6 mln Mg globally, whereas in Europe it is about 1.6 mln Mg. Most of these plastics are produced from polyethylene (PE) and polypropylene (PP) [5]. Many plastic materials used in plant and animal production are considered single use. This causes significant quantities of waste, which need to be managed. According to the APE Europe in 2019 about 63% of agri-plastic non-packaging waste was collected, whereas the remaining 37% was not identified [8]. It is speculated that this waste could be stored, burnt, buried or collected through local municipal or other waste streams [9]. It has to be pointed out that recycling of this type of waste in many cases is not feasible due to logistic and economic barriers. These barriers primarily include: high costs associated with the processing of waste prior to recycling caused by contamination by soil and plant residues (e.g., the contamination of plastic mulching films can range from 30% to 40% of the total mass of mulching films) and the low quality of the recyclate [9,10]. In reality, the plastic waste from plant and animal production is stored, landfilled or incinerated.

Therefore conventional, non-biodegradable, fossil-derived plastics should be phased out from agricultural applications and replaced with biodegradable and bio-based alternatives. However, not all of these plastic materials used in plant and animal production can be easily replaced. We analyzed the production of tomatoes in a greenhouse, in particular the use of plastics such as stem and arch support clips, and concluded that these conventional clips could be easily replaced with biodegradable and bio-based alternatives, which can be disposed of with plant residues through composting [11,12,13].

The annual production of tomatoes is estimated at 180 mln tons. It also generates significant quantities of biodegradable waste, including leaves, stems and damaged tomatoes. For example, greenhouse cultivation of tomatoes can generate up to 4.5 Mg of tomato leaves per 1 ha per day. Harvesting of tomatoes is associated with an annual generation of 15 Mg of plant residues per 1 ha [14]. Growing tomatoes requires the use of plant accessories such as stem and arch support clips. Assuming that one tomato plant needs a minimum of three stem support clips and six arch support clips, the mass of used clips from 1 ha is estimated at 0.644 Mg (in greenhouse cultivation) [13]. Biodegradable and bio-based clips could be managed through composting with the collected plant residues and other on-farm biowaste. Therefore, developing biodegradable and bio-based plant plastic accessories and replacing the conventional plastics from plant production could be a solution to reduce the consumption of fossil fuels and prevent microplastic contamination. 

In our previous work [11] we investigated the potential of using wood-derived and sewage sludge-derived biochars as fillers in Polylactic Acid (PLA) and Bioplast GS2189 biocomposites. Biochar is a stable and carbon-rich material obtained from plant or animal biomass through pyrolysis. Depending on the type of the substrate and pyrolysis parameters, biochars can be engineered to obtain specific properties for selected applications [15]. The most studied applications include using biochars for agriculture, soil remediation, the removal of contaminants from water and wastewater, and also the production of plastics [16]. Biochar can be used as a filler to produce biodegradable and bio-based plastics [17]. The previous study [11] reports the results from the laboratory analysis of the testing samples (i.e., the samples of biocomposites manufactured on a laboratory scale), including water adsorption, tensile strength, impact strength, differential scanning calorimetry (DSC), dynamic mechanical analysis (DMA) and optical and scanning electron microscopy (SEM). The results proved that biochar-added biocomposites can be used to produce agricultural accessories such as stem support clips and arch support clips for growing plants, e.g., tomatoes. Building on these results, we manufactured prototypes of stem and arch support clips from wood-derived biochar and Bioplast GS2189 (biodegradable bio-based polymer) and subjected them to laboratory analyses, field trials in the greenhouse cultivation of tomatoes and laboratory composting as the end-of-life management. 

The novelty of this work is demonstrated by the overall approach to the researching, developing, manufacturing and testing of bio-based and biodegradable alternatives to plant clips produced from fossil-derived and non-biodegradable materials such as polypropylene (PP) or polyvinyl chloride (PVC). Most of the literature references report the results from laboratory studies on biochar-added composites [17,18,19]. However, this work reports the selected properties and performance of the manufactured products, i.e., stem and arch support clips, validated and demonstrated in a relevant environment. It has to be emphasized that plant accessories such as stem and arch support clips are considered as single use. One of the suggested end-of-life practices is the composting of these biodegradable alternatives with selected organic waste (e.g., on-farm plant and animal residues, biomass, kitchen waste, etc.). Therefore, this study also investigated the composting of the manufactured clips with the selected on-farm organic waste as the end-of-life management method. 

The overall goal of this study was to manufacture biodegradable biochar-added and bio-based clips, and to analyze and test their selected properties in the greenhouse cultivation of tomatoes. The scope of the work included: (1) the production of stem and arch support clips in a pilot installation using injection molding technology, (2) an analysis of their biodegradability, disintegration and phytotoxicity, (3) an evaluation of the performance of the clips in the greenhouse cultivation of tomatoes and (4) an evaluation of the composting of the clips with on-farm organic waste as an end-of-waste management method. 

## 2. Materials and Methods

### 2.1. Substrates

Bioplast GS2189 (manufactured by BIOTEC, Pathum Thani, Thailand) was used as a base biopolymer and wood-derived biochar was used as a filler. The polymer was purchased from a commercial supplier (Noweko sp. z o.o., Bielsko-Biała, Poland), whereas wood-derived biochar was obtained free of charge from a commercial manufacturer (Fluid S.A., Sędziszów, Poland). Bioplast GS2189 is a plasticizer-free thermoplastic bio-based material. It is easy-flowing and, thus, suitable for processing by injection moulding to produce various completely biodegradable items [20]. Bioplast GS2189 is durable but it undergoes biodegradation (industry composting) according to the EN 13432 standard. Bioplast GS2189 is certified with “Ok compost” and certified compostable by VINCOTTE (Vilvoorde, Belgium). Wood-derived biochar is a plant-based carbon rich material with interesting properties that allow a wide range of applications in agriculture (e.g., soil enhancer) and environmental engineering (e.g., sorbent). It can also be applied as a filler to produce bio-based and biodegradable plastics to be used, e.g., in horticulture. Selected properties of wood-derived biochar used in this study are presented in Table 1.

**Figure 1:**
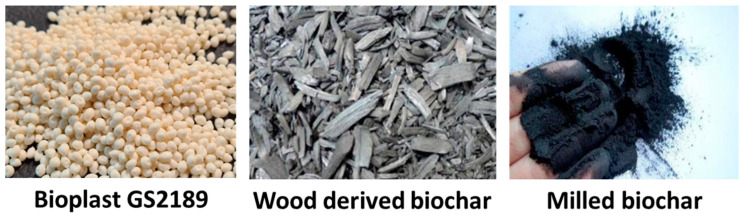
Bioplast GS2189 granules and wood-derived biochar.

Figure 1 presents substrates used for manufacturing of the stem and arch support clips.

Prior to manufacturing, the polymer granules were dried for 2 h at 60 °C in a laboratory drier (CD9, Shini), whereas wood-derived biochar was dried for 3 h at 95 °C in a drier (SNOL LSM01, Lithuania). The polymer and biochar were mixed in the ratio of 95:5 (by volume). To assure proper homogenization and even distribution of biochar, the polymer was immersed in thin Castrol oil film.

### 2.2. Stem and Arch Support Clips 

**Figure 2:**
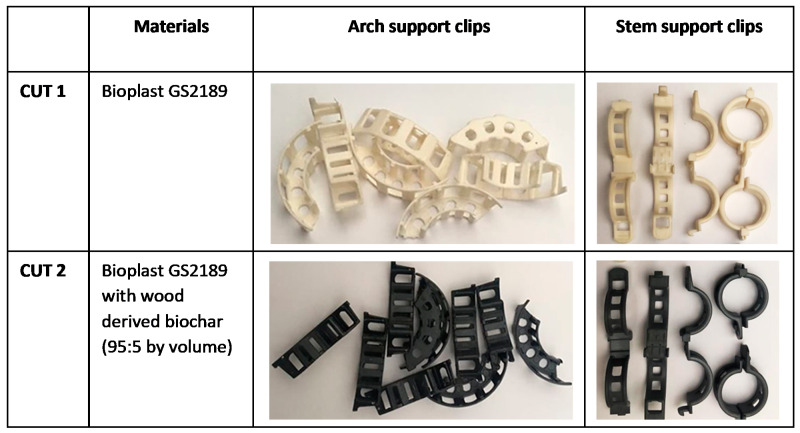
Arch and stem support clips.

Two types of plant accessories, i.e., stem and arch support clips (Figure 2) were manufactured: (1) from Bioplast GS2189 (referred to as CUT 1: CUT is the acronym for Częstochowa University of Technology where these clips were developed) and (2) Bioplast GS2189 mixed with wood-derived biochar as a filler (95:5 by volume) (referred to as CUT 2).

### 2.3. Methods

#### 2.3.1. Manufacturing of the Clips

The stem and arch support clips were manufactured in the technological laboratory at the Department of Technology and Automation (Częstochowa University of Technology, CUT) through injection molding with the injection molding machine (Krauss Maffei KM65 C3, München, Germany). Manufacturing was performed with the assistance of the industry partner. Injection molding was performed at 190 °C with the use of specialized molds for stem and arch support clips [11,12]. The average weights of a single stem support clip and arch support clip were 0.9 g and 2.22 g, respectively. 

#### 2.3.2. Analysis of the Selected Properties of the Clips

Laboratory analyses. The stem and arch support clips were subjected to a number of laboratory tests including: chemical analysis, biodegradability, disintegration and phytotoxicity (results presented for the stem support clips), field trials in greenhouse cultivation of tomatoes, laboratory and home composting. Chemical analysis, biodegradability, disintegration and phytotoxicity were performed in the external laboratory (Laboratory of Biodegradation and Microbiological Analysis, Institute of Biopolymers and Chemical Fibers, Łukasiewicz Research Network, Poland) as a part of the product certification process. Chemical analysis was performed according to the NL-13 procedure (developed at Laboratory of Biodegradation and Microbiological Analysis, Institute of Biopolymers and Chemical Fibers, Łukasiewicz Research Network, Poland) for As, Cd, Mo, Se (ETAAS), Cr, Cu, Ni, Pb, Zn (FAAS), Hg (CV AAS) and F (PN-EN ISO 10304-1:2009+C:2021). Biodegradation in home composting (“Home compost”) and industrial composting was performed according to the research procedure No. 6 on “Determination of the degree of decomposition of natural and synthetic raw materials as well as materials for various purposes in simulated home composting conditions on a laboratory scale. Determination of weight loss was performed based on the standards: PN-EN 14045:2021; PN-EN 14306:2010 and PN-EN ISO 20200:2016-01; PN-EN 13432:2002, PN-EN 14995:2009). Biodegradation in home composting was run at temperature of 30 °C for 48 weeks, whereas in industrial composting it was run at temperature of 58 °C for 12 weeks. Disintegration in industrial and home composting was conducted according to PN-EN 14995:2009 “Plastics—Evaluation of compostability—Test scheme and specifications”. Phytotoxicity was performed according to OECD 208 for the substrates after biodegradation of the clips (home and industry composting) with mustard, wheat and cuckooflower.

Greenhouse trial. Testing of the CUT 1 and CUT 2 stem and arch support clips was performed during cultivation of tomatoes in a greenhouse (at a private grower’s greenhouse located near Częstochowa, Poland). The clips were used to facilitate the growth of the plants by providing additional support to stems and preventing tomato trusses from dropping and breaking. The average number of the stem and arch support clips was 3 and 6 per 1 plant, respectively. This resulted in 2160 stem support clips and 4320 arch support clips for the tomato cultivation in a greenhouse of 135 m^2^ where 720 plants were grown. The clips were evaluated as alternatives to conventional, non-biodegradable, fossil-derived clips. In particular, the clips were subjected to observations regarding any changes to mechanical damage, color and durability. After tomato harvesting the plant residues with the clips were collected from the plots in the greenhouse and prepared for laboratory composting. 

Laboratory composting. Laboratory composting of the CUT 1 and CUT 2 stem and arch support clips was performed in the composting system consisting of two identical 60 L composting reactors with forced aeration, air flow and temperature control, and a set for collecting leachate and condensate (at the laboratories at Faculty of Infrastructure and Environment, Częstochowa University of Technology, Poland). The detailed description of this system is provided in our previous works [21,22]. Tomato plant residues were mixed with wheat straw and poultry manure in selected ratios (Table 2) and transferred into composting reactors 1 and 2 (treated as replications). The air flow rate was set at 35 L h^−1^. Prior to and after composting, the mixtures were analyzed for bulk density, moisture and organic matter, total organic carbon, nitrogen and pH [11,21,22,23].

The stem support (10 pieces) and arch support (10 pieces) clips were evenly distributed in the composting mixtures. The composting process was controlled by daily temperature measurements. In addition, carbon dioxide and ammonia were analyzed, and leachate and condensate were collected (results not presented here). Laboratory composting was run over 16 weeks. After this time the clips were withdrawn from the composting reactors, washed and dried at the temperature of 40 °C for 48 h in the SANYO STERILIZER MOV-212S (Sanyo Electric Co. Ltd., Osaka, Japan). Then, the clips were weighed to determine the weight loss due to degradation during laboratory composting.

#### 2.3.3. Statistical Analysis

Standard deviation was calculated for the results from laboratory tests and the weight losses of the investigated clips during composting.

## 3. Results 

### 3.1. Chemical Analysis of the CUT2 Stem Support Clips

The CUT 2 stem support clips (with the addition of biochar) were analyzed for selected trace elements. The results showed the presence of some of the trace elements, but these concentrations did not exceed the permissible values (Table 3). 

The chemical analysis of the CUT 2 stem support clips was a part of the product certification procedure.

### 3.2. Biodegradation and Disintegration of the CUT 2 Stem Support Clips under Composting Conditions

**Figure 3:**
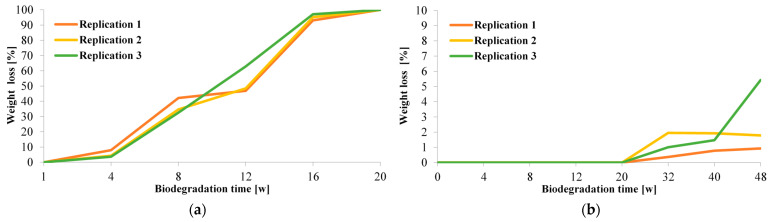
Weight loss of the CUT 2 stem support clips resulting from biodegradation during (**a**) 20-week industrial composting (at temperature of 58 °C) and (**b**) 48-week home composting (at temperature of 30 °C).

Biodegradation and disintegration in industrial and home composting were performed for the CUT 2 stem support as a part of the product certification process. Biodegradation of the CUT 2 stem support clips in industrial and home composting is presented in Figure 3. 

Industrial composting (at a temperature of 58 °C) resulted in 100% degradation of the CUT 2 clips after 20 weeks. In the case of home composting (at a temperature of 30 °C), degradation was slow, and after 48 weeks the maximum weight loss was 5.43%.

Disintegration of the CUT 2 stem support clips during industrial (58 °C, 12 weeks) and home (30 °C, 24 weeks) composting is presented in Table 4.

These results demonstrate that the higher temperature during industrial composting caused degradation and fragmentation of the clips into particles smaller than 2 mm.

### 3.3. Phytotoxicity 

**Figure 4:**
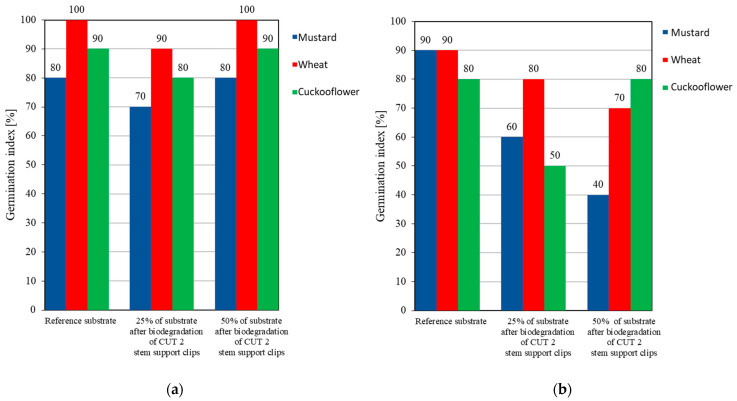
Germination index for the substrate after biodegradation of CUT 2 clips during (**a**) industry composting (at temperature of 58 °C) and (**b**) home composting (at temperature of 30 °C).

Phytotoxicity tests were performed for the substrates from the industrial and home composting of the CUT 2 stem support clips (Figure 4).

Phytotoxicity tests allow determination of the potential threat to the environment resulting from the contamination of soil with biodegradable plastic waste. The results show that the CUT 2 stem support clips did not affect the growth of the selected plants on the substrates from industrial and home composting. 

### 3.4. Greenhouse Trial

**Figure 5:**
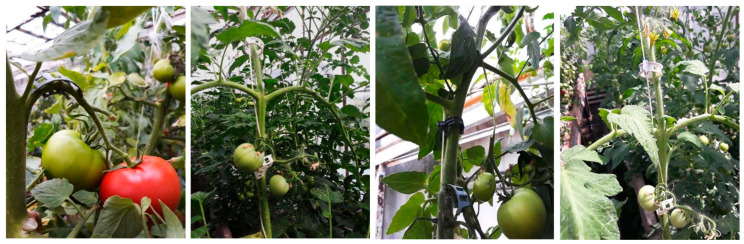
Stem and arch support clips (CUT 1 and CUT 2) applied to tomatoes in a greenhouse trial.

Biodegradable and bio-based CUT 1 and CUT 2 stem and arch support clips used in growing tomatoes in a greenhouse during a vegetation season proved to be suitable alternatives to the non-biodegradable clips produced from fossil-derived plastic (Figure 5). The CUT 1 and CUT 2 were easy to use and did not undergo any visual damages during the season. After tomato harvesting, the plant residues were subjected to laboratory composting. 

### 3.5. Laboratory Composting 

Laboratory composting was performed for the CUT 1 and CUT 2 stem and arch supporting clips over 16 weeks. After the completion of the process, weight loss was determined.

#### 3.5.1. Composting Process

**Figure 6:**
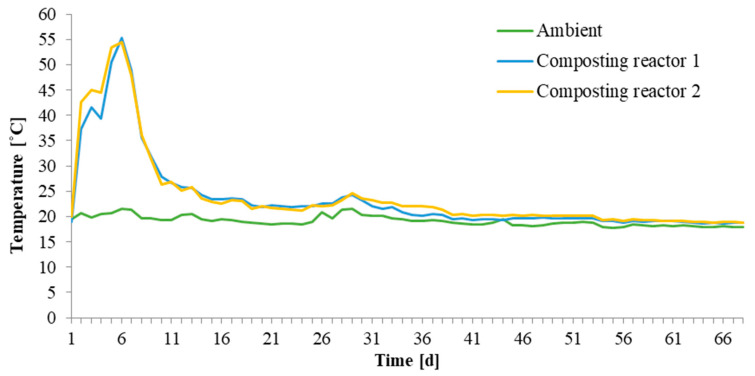
Evolution of temperature during the 16 week laboratory composting (data presented for 9 weeks).

Laboratory composting was monitored by daily temperature measurements (data presented for about 9 weeks). In the first week, a typical increase in temperature was observed, and then with time the temperature decreased and reached the ambient level (Figure 6).

#### 3.5.2. Weight Loss of Stem and Arch Support Clips during Laboratory Composting

During laboratory composting, the CUT 1 and CUT stem and arch support clips underwent degradation. The weight loss after 16 weeks is presented in Table 5. 

**Figure 7:**
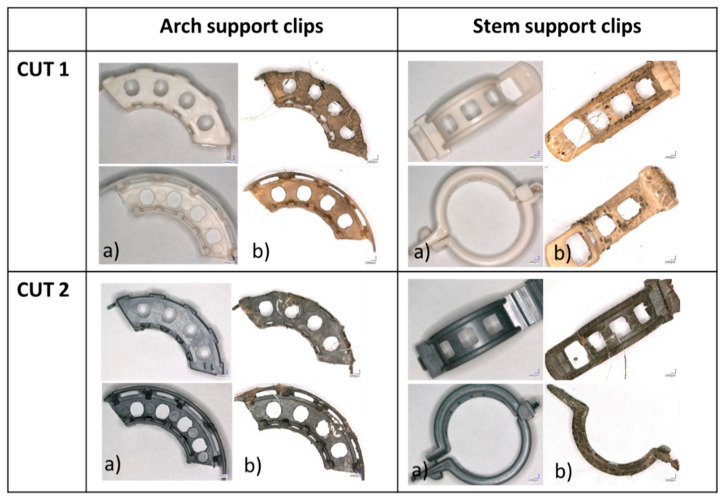
CUT 1 and CUT 2 arch and stem support clips prior to (**a**) and after the 16 week laboratory (**b**) composting (magnitude ×10).

The changes in the clips were analyzed under the microscope (magnitude ×10) and are presented in Figure 7.

#### 3.5.3. Composting Mixtures

The composting mixtures were analyzed prior to and after laboratory composting. Selected parameters are presented in Table 6.

The composting mixtures demonstrated suitable parameters for composting. The initial moisture content was about 67–69% with a C:N ratio of 15:1.

## 4. Discussion

### 4.1. Selected Properties of Biodegradable, Biochar-Added and Bio-Based Clips

The CUT 1 and CUT 2 stem and arch support clips were developed as alternatives to fossil-derived, non-biodegradable clips typically applied in tomato cultivation [24]. The CUT 1 clips were produced from biodegradable polymer, whereas the CUT 2 clips contained the addition of wood-derived biochar (as a filler). The mechanical properties of these clips were analyzed in our previous work [11]. In this work we focused on other properties of the biochar-added clips, in particular, chemical composition (i.e., heavy metals) biodegradability in home and industrial composting, disintegration (compostability) and phytotoxicity. These tests are essential for certification of the clips as compostable and biodegradable. The CUT 2 stem support clips (with the addition of biochar) decomposed at 100% during industrial composting (58 °C) after 20 weeks. However, during home composting (30 °C) these clips degraded only partially, and after 48 weeks the maximum weight loss was 5.43%. Disintegration of the clips during industrial composting resulted in 100% fragmentation into particles with sizes less than 2 mm. According to the requirements the particle size should be less than 10 mm. Composting conditions, in particular temperature, are crucial for the biodegradation of biodegradable materials. Phytotoxicity tests demonstrated that the substrates after industrial and home composting of the CUT 2 stem support clips did not have a negative effect on the growth of the test plants (i.e., mustard, wheat, cuckooflower). 

Phasing out non-biodegradable, fossil-derived plastics from agricultural and horticultural applications and replacing them with bio-based and biodegradable materials is highly recommended. However, bio-based and biodegradable plastics can have limited applications due to their properties and consumer needs and expectations. The challenge is to develop bio-based and biodegradable plastic materials with similar properties to conventional plastics that would undergo rapid degradation in the natural environment [25]. Developed clips were only tested for biodegradation in composting. Therefore, additional tests on the CUT 1 and CUT 2 stem and arch support clips should also include tests for degradation in soil and water environments.

### 4.2. Performance of the Clips in the Greenhouse Trial

The CUT 1 and CUT 2 stem and arch support clips were tested in greenhouse tomato cultivation. According to the observations, these clips performed in a similar way to the conventional fossil-derived and non-biodegradable clips. During the growing season the clips did not demonstrate any visible mechanical changes. After the completion of the growing season and harvesting of tomatoes, the plant residues (i.e., leaves, stems and damaged tomatoes) were collected and used for laboratory composting. In the case of biodegradable clips they do not need to be removed from the plant residues and can be disposed of through composting with, e.g., on-farm biowaste. These clips can also be applied in the cultivation of other edible plants to provide support. The costs of single use biodegradable and bio-based materials in growing tomatoes in a greenhouse, including arch and stem support clips as well as biodegradable mulching film, are comparable to the use of conventional plastics. Such cost estimation was performed by Pudełko [13], and included: the cost of purchasing biodegradable plastic clips, the costs of on-farm handling and managing of waste materials after tomato harvesting (i.e., labor) and the costs of disposal by a specialized company. Taking these costs into account, the overall cost of the use of conventional plastic clips in growing tomatoes can be comparable or lower than the cost of biodegradable and bio-based clips [13].

### 4.3. End-of-Life Management of the CUT 1 and CUT 2 Stem and Arch Support Clips through Composting

Laboratory composting of the CUT 1 and CUT 2 stem and arch support clips was performed in order to test composting as an end-of-life management. The results demonstrated that under the specific conditions (i.e., the composition of the composting mixture, moisture content, C:N ratio, temperature and air flow rate) these clips did not undergo complete degradation during 16 week laboratory composting. However, the average weight loss of the CUT 1 and CUT 2 arch support clips was 27% and 20%, respectively. As for the CUT 1 and CUT 2 stem support clips, the average weight loss was 32% and 14%, respectively. These differences could have resulted from the size and shape, the weight and the composition of the clips. For example, the average weight loss for the CUT 1 arch support clips was higher by about 7% in comparison to the CUT 2 arch support clips, which could have been due to the addition of biochar. Similar observations were reported by [19] who concluded that the addition of 15% (by weight) to PLA/PBA resulted in an extension of the degradation time. Biochar can function as a reinforcement due to its porous structure [18]. Additionally, the weight of the stem and arch support clips differed (0.9 g and 2.22 g, respectively), which had an effect on degradation during composting. Composting is a complex process and is influenced by specific conditions. It has to be emphasized that compositing mixtures can be very heterogenic and single clips could have been exposed to slightly different conditions within the same process [26]. However, these conclusions need further confirmation.

The degradation rates of plastics in the environment have been studied by many researchers [3,26] who analyzed different factors affecting degradation rates, including environmental such as moisture, heat, light and microbial activity. Degradation of some biodegradable plastic materials in the environment can be less efficient, e.g., in fresh and salt water than in soil or compost. However, some biodegradable plastics, e.g., biodegradable foils, under realistic conditions can persist in the environment for several years. It has not been concluded whether these biodegradable plastic materials in different environments undergo mineralization or just disintegrate into smaller particles [27]. Therefore, laboratory composting of the CUT clips should be validated under realistic conditions, e.g., in a composting facility. The obtained compost should be tested to assure that it does not contain biodegradable plastic particles and can be safely applied to soil. 

## 5. Conclusions and Prospects

With reference to the obtained results, the following conclusions were formulated:

(1) The biodegradable biochar-added and bio-based plastic accessories, i.e., CUT 2 stem support clips proved to be fully degradable in industrial composting (at 58 °C). They did not demonstrate phytotoxicity after industrial and home composting towards selected plants. However, CUT 2 stem support clips did not degrade in home composting (at 30 °C). Disintegration of these clips occurred during industrial composting, whereas it did not occur during home composting.
(2) The CUT 1 and CUT 2 stem and arch support clips performed similarly to conventional clips (manufactured from, e.g., PP or PVC) during tomato cultivation in a greenhouse and proved to be satisfactory alternatives to conventional non-biodegradable fossil-derived clips.
(3) The end-of-life management of stem and arch support clips through laboratory composting demonstrated that the clips underwent partial degradation. In the case of CUT 2 clips (with the addition of biochar), the weight loss was lower than in the case of CUT 1 clips.
(4) Degradation of the biodegradable and bio-based plant clips during composting can depend on the size, weight and thickness of these clips.

Future work should focus on the end-of-life management of biodegradable accessories for growing plants. In particular, more work is needed to determine the optimal conditions for the composting of biodegradable clips with on-farm animal and plant residues in realistic conditions, e.g., in windrow composting. This should result in the rapid degradation of biodegradable clips to assure that no biodegradable plastic particles are present in the compost. 

## Figures and Tables

**Table 1:** Selected properties of wood-derived biochar [11,13].

Properties	
Moisture content (%)	5.25
pH	7.4
Ash (%)	4.13
Conductivity (µS cm^−1^)	169 ± 15
Total carbon (%)	81.4 ± 3.4
Total organic carbon (%)	75.3 ± 7.2
Calorific value (kJ kg^−1^)	29,780
Particle size (mm)	<1.5

**Table 2:** Composition of the composting mixtures.

	Composting Reactor 1	Composting Reactor 2
Poultry manure–tomato plant residues–wheat straw (by mass), %	1:2:0.2	1:2:0.2
Total weight (wet basis), kg	13.22	14.65

**Table 3:** Chemical analysis of the CUT 2 stem support clips.

Elements	Concentration mg kg^−1^ Dry Matter	Permissible Concentration mg kg^−1^ Dry Matter
Zn	3.42 ± 0.52	150
Cu	<1	50
Ni	<1	25.0
Cd	0.030 ± 0.008	0.5
Pb	<1.5	50
Hg	<0.01	0.5
Cr	<1	50
Mo	<0.2	1
Se	<0.1	0.75
As	<0.1	5
F	<10	100

**Table 4:** Disintegration of the CUT 2 stem support clips under industry (at temperature of 58 °C) and home (at temperature of 30 °C) composting conditions.

	Particle Size > 2 mm	Requirement
Industry composting	0%	<10%
Home composting	100%	<10%

**Table 5:** Changes in the average weight of the CUT 1 and CUT 2 stem and arch support clips prior to (initial) and after laboratory composting (final).

	Arch Support Clips		Stem Support Clips	
	Average Weight, g		Average Weight, g	
	Initial	Final	Initial	Final
CUT 1	0.92 ± 0.01	0.67 ± 0.12	2.30 ± 0.02	1.56 ± 0.84
CUT 2	0.92 ± 0.01	0.74 ± 0.11	2.26 ± 0.09	1.84 ± 0.29

**Table 6:** Characteristics of the composting mixtures prior to and after laboratory composting.

Parameter	Prior to Composting		After Composting	
	Reactor 1	Reactor 2	Reactor 1	Reactor 2
Bulk density, kg m^−3^	244.81	271.30	150.0	235.19
pH	7.75	7.66	9.02	8.66
Conductivity, ms cm^−1^	5.56	8.22	9.47	11.45
Moisture content, %	67.26	69.00	58.99	78.32
Organic matter, %	78.90	79.35	73.58	70.70
Total organic carbon, %	43.84	44.08	40.88	39.28
Nitrogen, %	2.88	2.95	3.22	3.00
C:N	15:1	15:1	13:1	13:1

## Data Availability

Not applicable.
